# Management of valgus knee with irreducible patellar dislocation and MCL rupture: A case series

**DOI:** 10.1016/j.ijscr.2019.06.018

**Published:** 2019-06-16

**Authors:** Sholahuddin Rhatomy, Hendri Purnama, Charanjeet Singh, Riky Setyawan, Dwikora Novembri Utomo

**Affiliations:** a Department of Orthopaedics and Traumatology, Dr Soeradji Tirtonegoro General Hospital, Klaten, Indonesia; b Faculty of Medicine, Public Health, and Nursing, Universitas Gadjah Mada, Yogyakarta, Indonesia; c Fellowship of Hip and Knee, Orthopaedics and Traumatology Department, Dr. Soetomo General Hospital Surabaya, Indonesia; d Department of Orthopaedics and Traumatology, Gleneagles Hospital Kuala Lumpur, Malaysia; e Soeradji Tirtonegoro Sport Center and Research Unit, Dr Soeradji Tirtonegoro General Hospital, Klaten, Indonesia; f Department of Orthopaedics and Traumatology, Dr. Soetomo General Hospital Surabaya, Indonesia

**Keywords:** Valgus knee, Patellar dislocation, Medial patellofemoral ligament (MPFL), Q angle

## Abstract

**Introduction:**

The high rate of recurrence in patellar dislocation treatment, requires a more comprehensive action, this is due to causes not only single but often multifactorial, including problems with static stability, dynamic stability or both.

**Presentation of case:**

1^st^ case: A-39-years-old male, complain of irreducible right patella dislocation with valgus knee and already done soft tissue procedure for patella dislocation. Long-leg radiographs of the right leg showed 18° valgus mechanical angle. 2^nd^ case: A-26-years-old obese female, complain of dislocation of left patella and history of surgery for dislocation at 5 years old. Long-leg radiographs of the right leg showed 11° valgus mechanical angle.

**Discussion:**

After knowing the cause of the patellar dislocation from history taking, physical and supporting examination, we performed lateral open wedge distal femoral osteotomy also MPFL and MCL reconstruction, and tibial tuberosity medialization osteotomy. There is improvement mean score in Tegner Lysholm Knee Scoring system and IKDC Scoring at 6 months after surgery.

**Conclusion:**

Lateral open wedge distal femur osteotomy combine with MPFL and MCL reconstruction and tibial tuberosity medialization realignment procedure can be successfully done for improve irreducible patellar dislocation in valgus knee, from clinical and radiological evaluation have good outcome after surgery.

## Highlights

• Lateral open wedge distal femur osteotomy.
• With MPFL and MCL reconstruction.
• Tibial tuberosity medialization realignment procedure.
• This procedure can improve irreducible patellar dislocation in valgus knee.

## 1 Introduction

Irreducible patellar dislocation is a rare case where the patella dislocates during flexion and extension of the knee, and it usually presents with pain or swelling. The incidence considerably higher in children and adolescents aged between 10 and 19 years with an incidence of 31 per 100,000 [1,2].

The etiology of patella dislocation is multifactorial and most common dislocates laterally caused by bony abnormalities (patella alta, lateral trochlear dysplasia, patella hypoplasia, lower limb malalignment, rotational abnormalities) or soft tissue pathology (vastus medialis obliquus atropy, medial retinaculum laxity, injuries to the MPFL, tight lateral structures, or ligamentous laxity) [4].

Valgus knee is a risk factor for lateral patellar instability. Valgus malalignment increases the Q angle, which contributes to lateralization and maltracking of patella. Q-angle is the angle formed by a first line drawn from the anterior superior iliac spine (ASIS) to the center of patella and a second line drawn from center of patella to tibial tubercle. Q-anle represents a risk factor for patellar instability. Increased Q-angle is thought to create excessive lateral forces on the patella through a bowstring effect. The Q-angle averages 14° in males and 17° in females. This value increased in presence of valgus knee, increased femoral anteversion, increased external tibial torsion, or a lateralized tibial tuberosity [4,14].

The radiographic evaluation of patients with patella instability begins with standard anteroposterior, lateral, axial view radiographic, CT Scan and MRI.

**Fig. 1:**
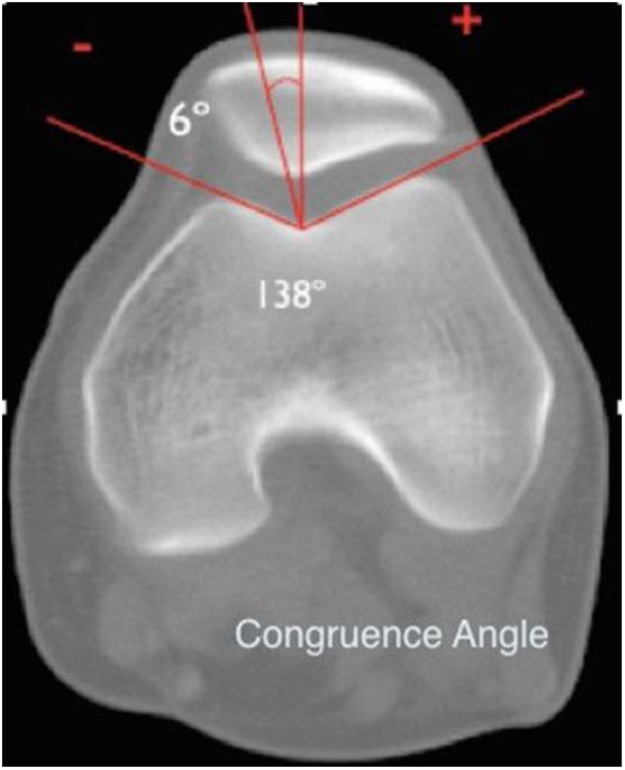
Merchant view (axial view) to measure congruence angle. [1] Bisect the angle of the femoral trochlea [2]. Draw a line from the apex of the femoral trochlea through the apex of the patella. The angle between these two lines is the congruence angle.

From radiographic Merchant view can measure groove angle and congruence angle (Fig. 1). Normally, the groove angle, which is formed by the highest points of the femoral condyles and the deepest point of the intercondylar groove, is about 138°. The congruence angle is obtained from drawing two lines. The first line is the bisection of the femoral Groove angle. The second line is drawn from the lowest point of the articular margin of the patella to the deepest point of the groove, the congruence angle average was −6° [4].

Various surgical techniques have been introduced to treat patellar dislocation in valgus knee, including a single procedure of bone or soft tissue, but the failure rate is high and often recurrence, here we use a combination of bone and soft tissue which results are much better.

**Fig. 2:**
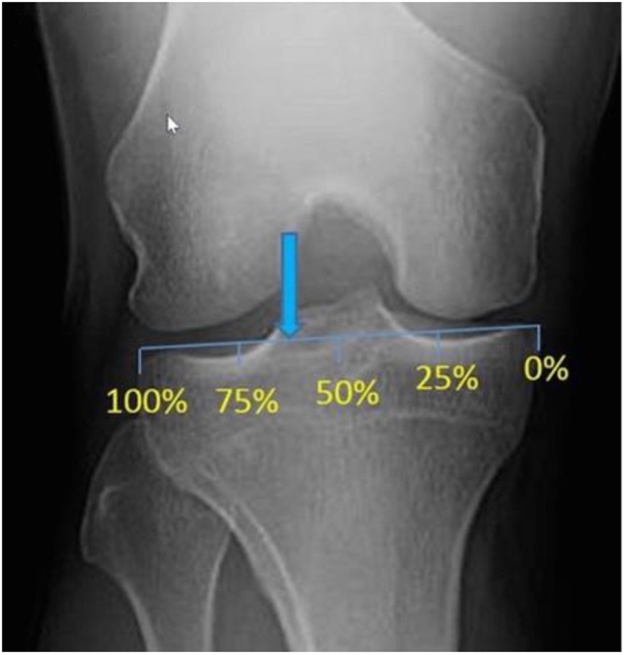
Fujisawa detemined a point (arrow) located at 62% of the tibial plateau width when measured from the medial tibial plateau.

To determine the angle of correction can use the research of Fujisawa et al. The weight bearing line should pass from 62% of the tibial plateau width when measured from the edge of the medial tibial plateau (Fig. 2) [5].

Nonoperative treatment is indicated for first-time dislocation, the immediate goals are to decrease the effusion, increase range of motion, and stimulate VMO activity. The rehabilitation program in patellar instability focuses on VMO strengthening, core stability improvement, full ROM restoration, and proprioception enhancing.

Operative treatment can be divided into soft tissue procedures (medial repair, medial imbrication, MPFL reconstruction, lateral release) and bony procedures (varus realignment distal femur osteotomy, tibial tuberosity transfers, trochleoplasty) [15].

Varus distal femur osteotomy is indicated in patients with irreducible dislocation patella with valgus knee and concurrent symptomatic MCL incompetence. Contraindications to the procedure include inflammatory arthritis, flexion contracture in 15° or greater, fixed valgus deformity in greater than 20°, knee flexion in less than 90°, and arthritis in the medial or patellofemoral compartments [7].

Distal realignment procedures are indicated in patients with Tibial Tuberosity-Trochlear Groove (TTTG) distance >20 mm (medialization of the tibial tuberosity) or patella alta (distalization of the tibial tuberosity) or both.

**Fig. 3:**
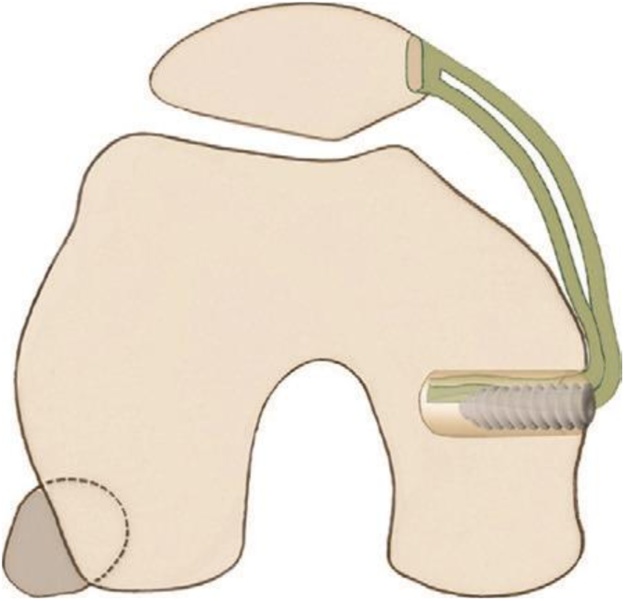
Schematic representation of medial patellofemoral ligament (MPFL) reconstruction. A two-strand gracilis tendon graft is passed in a loop through the patella and is fixated with an absorbable interference screw in the femur.

Several techniques MPFL reconstruction have been proposed with the use of different fixation methods and grafts (Fig. 3), but the overall principle remains the same. Our procedure of choice is reconstruction with gracilis tendon [8].

Treatment the patients with irreducible patellar dislocation with valgus knee is challenging, often fail and experience recurrence. We report a case of irreducible patellar dislocation with valgus knee treated with a corrective osteotomy of the distal femur combined with lateral release and medial reconstruction and distal patella realignment osteotomy procedure.

## 2 Material and methods

### 2.1 Case

**Fig. 4:**
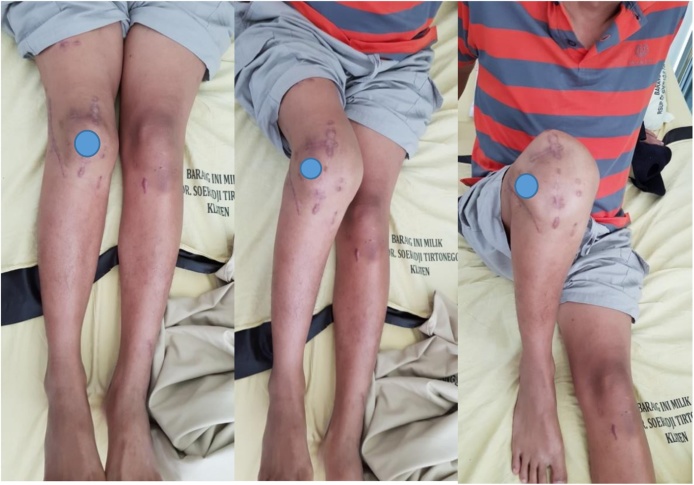
The clinical pictures lateral dislocation of right patella in extension, midflexion and flexion position.

**1^st^ case**: A-39-years-old male referred to our General Hospital with sensation of giving away and unable to extend on the right knee after car accident 1.5 years ago. He had history of close fracture of lateral condyle right femur. The patient complain of irreducible patellar dislocation during flexion and extension of the right knee joint. He underwent 4 times surgery of his right knee by other orthopaedic surgeon. The first surgery result still unreduced fragment fracture, and we done open reduction and internal fixation (ORIF) also PCL reconstruction. The right knee became valgus knee after fracture union and instability sensation on the medial knee joint. On physical examination, the right knee had 18° of valgus deformity, moderate instability to valgus stress, complete lateral dislocation of the patellae during extension and flexion, and tenderness at the lateral joint line (Fig. 4). Range of motion was 0–110°.

**Fig. 5:**
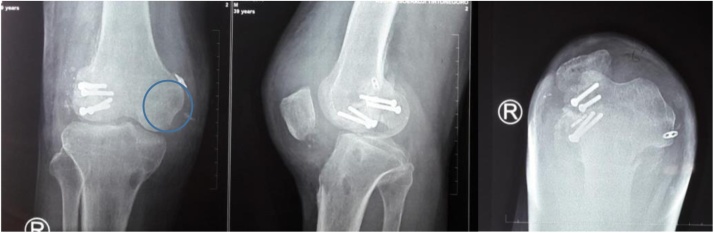
Anteroposterior, Lateral and Skyline view plain radiograph showed the lateral patellar dislocation.

During radiographic examination, the standing knee radiograph showed mild arthritic changes on the lateral compartment of the right knee that were not observed on the left knee. The long-leg weight-bearing standing radiograph showed 18° valgus on the right knee and 5° valgus on the left knee with depression of the lateral tibia plateau and a mechanical axis passing outside the lateral compartment, and the weight bearing line locates lateral to Fujisawa point. The anatomical lateral distal femoral angles of the right and left knees were 105° and 82° respectively (normal value is 81° and 62°, respectively); the medial proximal tibial angles were 94° and 93° respectively (normal value is 87° and 63°, respectively). This result confirmed valgus deformities of both knees. A skyline view radiograph showed complete lateral dislocation of the patella (Fig. 5) [9].

**Fig. 6:**
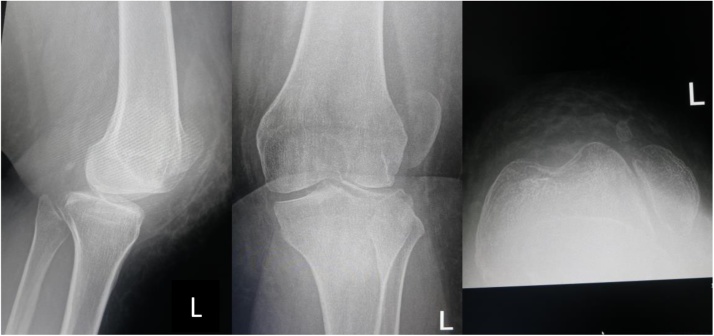
X ray AP, lateral and skyline view radiograph show the lateral patellar dislocation.

**Fig. 7:**
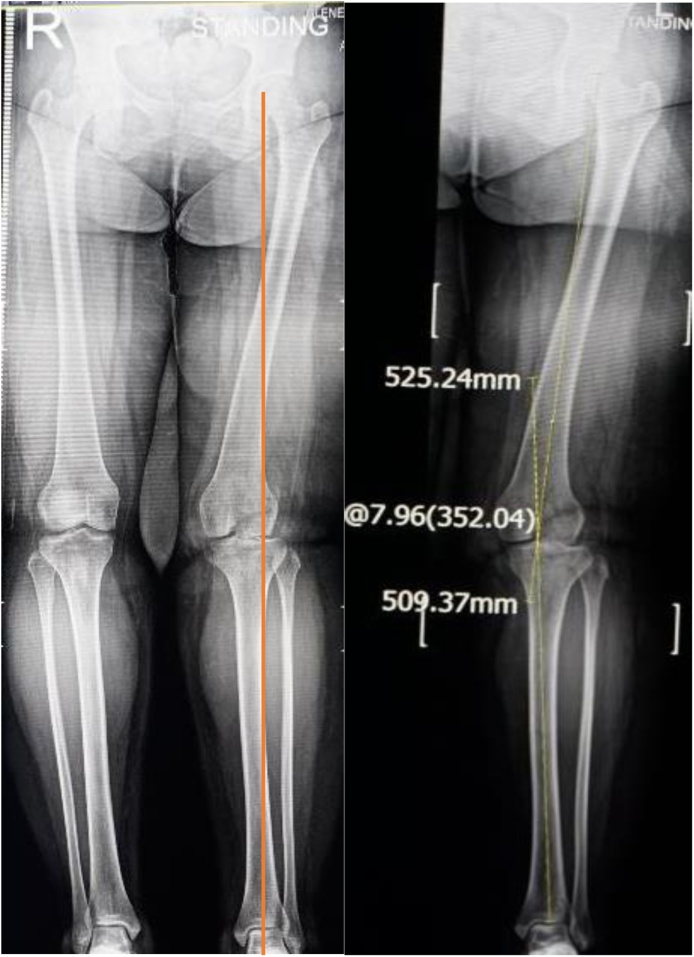
Long standing hip-ankle AP X-ray show the mechanical line angle 110 valgus of left lower extremity.

**Fig. 8:**
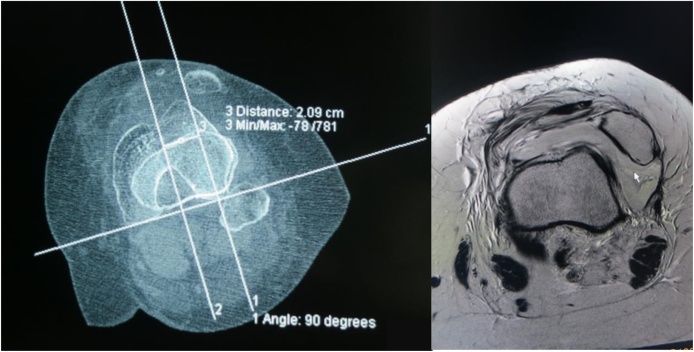
CT scan and MRI show dislocation of patella ‘.

**2^nd^ case**: A-26-years-old obese female came into outpatient clinic with chief complain of left patellar dislocation. She had history of left knee surgery for dislocation at age 5 years old. Her left knee never felt comfortable and she is frequent fall down. In physical examination, we found left knee valgus alignment, its Q angle was 25°, muscle atrophy and positive J sign. Long-leg radiographs of the left leg showed a mechanical angle of 11° of valgus, and the weight bearing line locates lateral to Fujisawa point (Fig. 6, Fig. 7, Fig. 8).

### 2.2 Methods

Written informed consent was obtained from the patient for publication of this case report and accompanying images. A copy of the written consent is available for review by the corresponding author of this journal on request. Our institutional review board also provide an ethical approval in the form of case series with KE/FK/1387/EC/2018 as the protocol number. This study has been registered in the publicly accessible database and having a unique identifying number: researchregistry4724. Patient had diagnosis confirmed before operation by clinical evidence alone. Scanogram, Skyline view X-ray and MRI were performed in this patients where the concomitant injuries were suspected. Both of two patients underwent the same surgical procedure by orthopaedic surgeon specialized in knee surgery. This research work has been reported in line with the PROCESS criteria [10].

Preoperative planning for combination of multiple surgery procedure is indicated for patients with irreducible patellar dislocation and concomitant valgus knee who have failed with non-operative management.

Patient had local anesthetic procedure using intravenous spinal anesthetic agents. Single dose prophylactic antibiotics were administered intravenous intraoperative. The patient laid in supine position on operating table. The site surgery leg placed tourniquet tools. A diagnostic arthroscopy was performed to see the patellar and trochlear articular cartilage, and ensure there is no contraindications to distal femoral osteotomy. It showed a total ruptured of the ACL and lateral patellar dislocation.

**Fig. 9:**
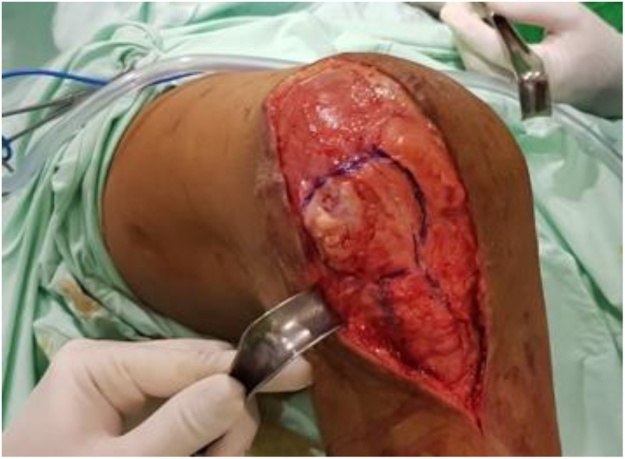
Longitudinal skin incision was made beginning 10 cm above the patella and distal extending to the apex of the patella.

First, a lateral open wedge osteotomy of the distal femur was performed. A longitudinal skin incision was made beginning 10 cm above the patella and distal extending to the apex of the patella (Fig. 9). We performed tibial tuberosity osteotomy, then the fascia was incised and the vastus lateral also medial muscle was stripped from the intermuscular septum and retracted proximally. A blunt retractor was passed over the femur to expose the anteromedial aspect of supracondylar area of the femur.

The bi-planar osteotomy began in the lateral supracondylar area and ended within the medial femoral condyle. The starting point for the distal osteotomy at the lateral femur was marked with electrocauter after temporary application of the plate. A guide pin was inserted under the fluoroscopy, and the osteotomy was created using an oscillating saw, followed by osteotomes. The osteotomy was stopped 10 mm before the medial cortex to preserve medial femoral cortex. The osteotomy was carefully opened about 1 cm by applying consistent traction. The axis alignment of the leg was evaluated using fluoroscopy. The osteotomy was stabilized using a Condylar butress plate 5 holes. After stabilization of the osteotomy, we filled the bone gap with allograft (Bovine). A lateral release was performed.

**Fig. 10:**
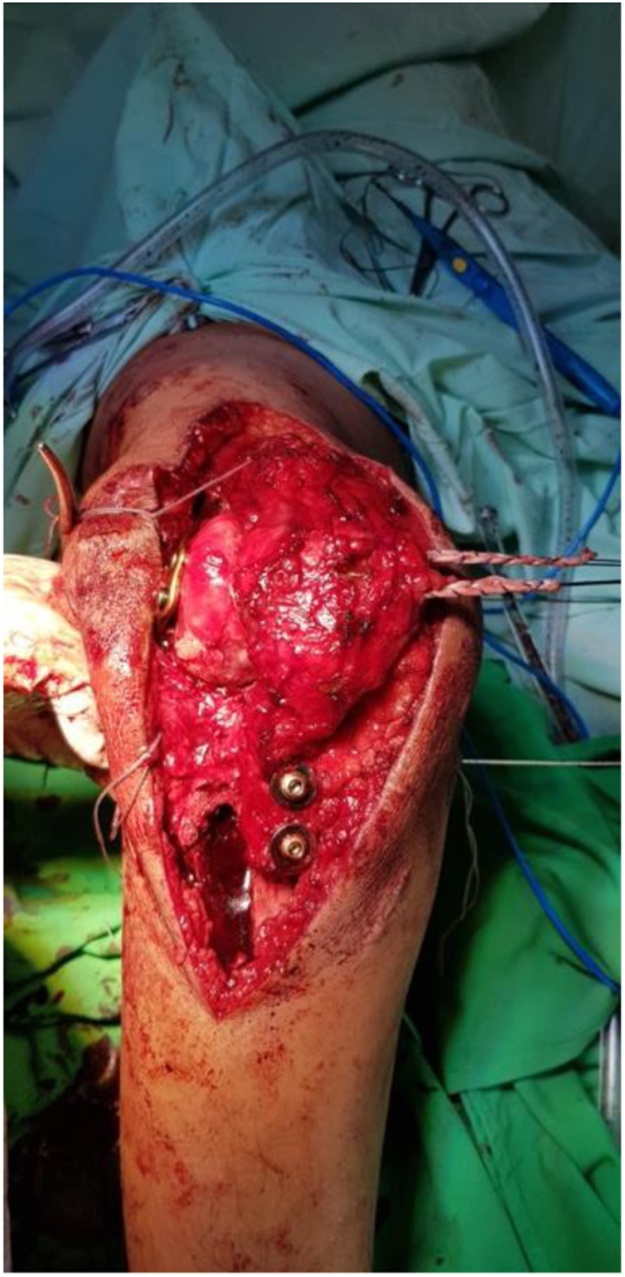
MPFL reconstruction with gracilis tendon autograft was looped through and medialisation tibial tuberosity.

Then, we did reconstruction for patellar MPFL insertion (Fig. 10). Two stab incisions in the medial retinacular sleeve directly adjacent to the medial patellar edge were made and a gracilis tendon autograft was looped through. A more medial incision of layer 1 was performed posterior to the medial epicondyle. The femoral attachment site can be referenced 10 mm proximal and 2 mm posterior from the medial femoral epicondyle [11]. The femoral end of the MPFL reconstruction was continued by creating a 6 mm bone tunnel starting at the native MPFL femoral insertion which was confirmed by fluoroscopic control. The free ends of the gracilis autograft was passed between the two layers of the medial patellar retinaculum and inside the femoral tunnel. The free end was fixed in the femoral tunnel together with MCL reconstruction using a bio screw at 60° flexion position, then finishing the MPFL reconstruction. Thus restoring proper tension in the medial ligament complex.

Semitendinosus autograft was prepared with creating a 7-mm diameter by 14-mm length to reconstruct the MCL. A 3-cm longitudinal skin incision was made over the medial femoral epicondyle. We inserted a guide pin at 10 mm proximal and 2 mm posterior to the medial femoral epicondyle and in a 15° anterior direction to avoid the intercondylar notch, same point with MPFL insertion. The location of the pin was confirmed with fluoroscopy. Proximal tibia anatomic insertion was estimated posterior to the pes anserinus insertion. Isometric movement was tested through knee motion from 0° to 90°, until the loop was isometric. We marked the isometric point on the tibia. We reamed over the guide pin with a 7-mm diameter reamer to a depth of 20 mm. We were inserted the semitendinosus tendon into the femoral socket and fixed with a bio screw together with MPFL reconstruction. The semitendinosus tendon tissue was passed under the skin and distally. We tensioned the graft and fixed it on the tibia. The MCL graft was tensioned with the knee at 20° flexion under varus stress and fixed at the isometric point on the tibia with a bioscrew. The subcutaneous tissue and skin were closed.

The tibial tubercle including the patellar tendon insertion, that had undergone osteotomy, was fixed 1 cm medial than original location using 2 cortical screws 36 mm with washer. Graft tensioning was performed in 70° of flexion.

The patellofemoral alignment was checked throughout the full range of motion, and lateral dislocation of the patella was no longer occurs.

**Fig. 11:**
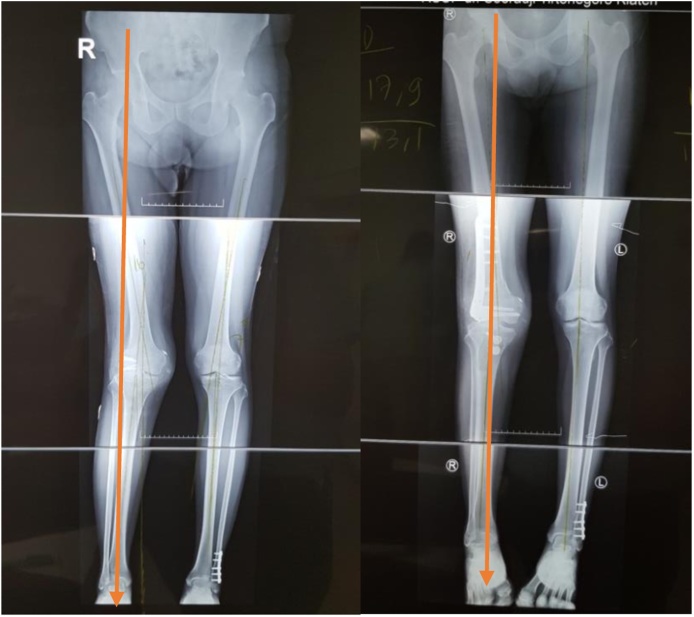
Mechanical axis intersection point on lateral to Fujisawa point before surgery(left side) and mechanical axis intersection point on Fujisawa point after realignment osteotomy(right side).

**Fig. 12:**
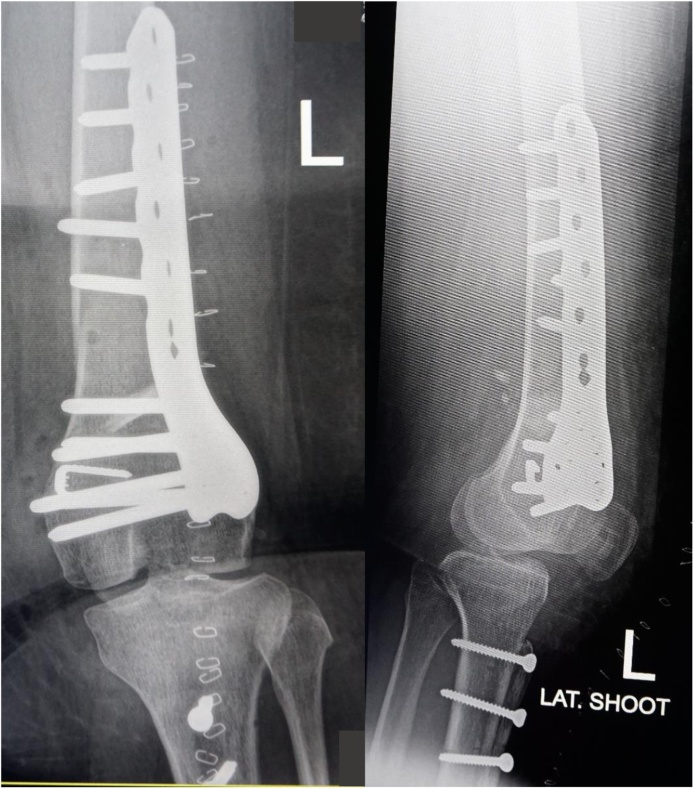
Skyline view radiograph show normal position of patella after reconstruction.

Post-operative long-leg radiographs (Fig. 11) and sky line view radiographs (Fig. 12) showed correction of the valgus deformity and no evidence of patellar dislocation. Patients did not suffer pain and patellar dislocation. Three day postoperatively, leg raising exercises with gradual flexion exercise was started. Full range of motion was obtained with no evidence of patellar instability at fourth week postoperative. Partial weight bearing was gradually permitted at sixth week postoperative, and standing hip to ankle radiographs and sky line radiographs are obtained 3 months after surgery to assess correction of deformity and full weight bearing was permitted at twelfth week postoperative, the patient may gradually return to activities as tolerated after 4 month. Six months after surgery we evaluate clinical functional score using IKDC, Tegner-Lysholm, and radiographs

## 3 Result and discussion

**Fig. 13:**
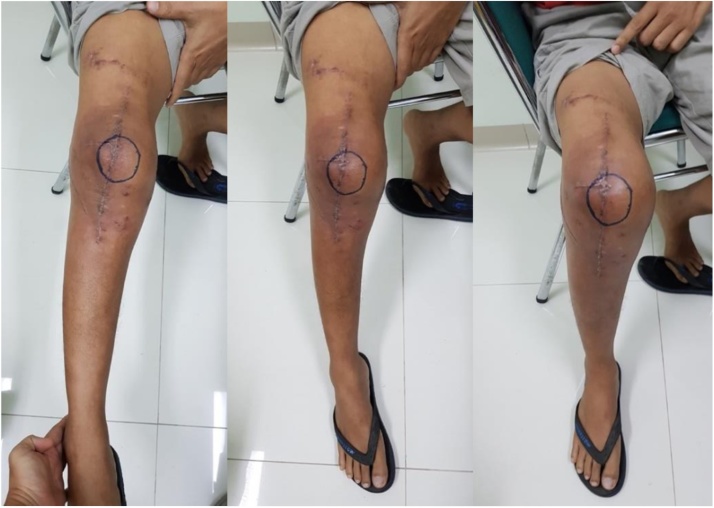
The clinical picture 6 months after surgery still normal position of right patella in flexion, midflexion and extension.

The result mean score Tegner Lysholm Knee Scoring system before surgery was 32 and 6 months post surgery score was 88. Mean score IKDC Scoring before surgery was 23 and 6 months post surgery was 78,2 indicating an excellent clinical result (Fig. 13).

The cause of patellar dislocation is multifactorial include the bone and soft tissue pathology. There are several techniques for treatment irreducible patellar dislocation, The clinical results of isolated lateral release for patellar instability, reportedly are poor, and include ongoing instability and poor patient satisfaction. Combination procedure, should be performed to achieve relatively satisfying results [10].

Realignment procedures can be performed additionally to improve the joint stability. They are broadly classified into bone and soft tissue proximal also distal realignment procedures. Proximal bone realignment technique are open or close wedge distal femoral osteotomy, proximal soft tissue realignment procedures including lateral release and MPFL reconstruction. Distal realignment bony procedures is tibial tuberosity transfers [10].

Proximal realignment is the most effective treatment for reduction of patellar dislocation. However, vastus medialis advancement can cause an increase in pressure on the knee, which eventually results in patellofemoral arthritis. Recently, medial patellofemoral ligament reconstruction using semitendinosus or gracillis tendon is recommended [10].

Coventry [9] reported that femoral supracondylar varus osteotomy should be performed in patients with ≥12° of symptomatic valgus knee or with ≥10° of tilt of the articular surface. Healy et al. [11] recommended to perform corrective osteotomy of the distal femur for ≥15° of valgus knee. Shen et al [13] suggested proximal soft tissue realignment and distal femoral osteotomy and anteromedial tibial tubercle transfer to treat valgus knee with ≥20° of femorotibial angle. Femorotibial angle was 16° in our patient. We thought proximal soft tissue realignment alone was not sufficient to realign the patella and performed additional femoral supracondylar osteotomy. Femoral supracondylar osteotomy for valgus knee can be performed using an open or closed technique, we used an open wedge osteotomy technique and took care not to damage the joint capsule during surgery.

We believe that clinical improvement of irreducible dislocation of the patella can be obtained with correction of soft tissue imbalance and realignment of the patellofemoral and tibiofemoral joints. In particular, soft tissue procedures should be followed by bony procedures including femoral supracondylar osteotomy in cases of irreducible dislocation of the patella with valgus knee.

## 4 Conclusion

Combine multiple procedure surgery with varus distal femur osteotomy, MPFL and MCL reconstruction, lateral retinacular release and distal patella realignment osteotomy can be successfully done with good outcome after surgery to prevent recurrent patellar dislocation in valgus knee with irreducible patellar dislocation.

## Conflicts of interest

No potential conflict of interest relevant to this article was reported.

## Sources of funding

The authors declare that this study had no funding resource.

## Ethical approval

The informed consent form was declared that patient data or samples will be used for educational or research purposes. Our institutional review board also provide an ethical approval in the form of case report with KE/FK/1387/EC/2018 as the protocol number. This study has been registered in the publicly accessible database and having a unique identifying number: researchregistry4724.

## Consent

Written informed consent was obtained from the all of the patients for publication of this case report and accompanying images. A copy of the written consent is available for review by the corresponding author of this journal on request.

## Author’s contribution

Sholahuddin Rhatomy, Charanjeet Signh, and Dwikora Novembri Utomo conceived the study. Sholahuddin Rhatomy and Hendri Purnama performed surgery, collected and analysed data, also drafted manuscript. Riky Setyawan drafted the manuscript, analysed data, and critically revised the manuscript for important intellectual content. Sholahuddin Rhatomy, Sharanjeet Signh, and Dwikora Novembri Utomo reviewed and edited manuscript. Sholahuddin Rhatomy and Riky Setyawan facilitated all project-related tasks.

## Registration of research studies

This study has been registered in the publicly accessible database and having a unique identifying number: researchregistry4724.

## Guarantor

Sholahuddin Rhatomy, M.D.

## Provenance and peer review

Not commissioned, externally peer-reviewed.
